# Studying effects of neuromuscular electrostimulation therapy in patients with dysphagia: which pitfalls may occur? A translational phase I study

**DOI:** 10.3205/000294

**Published:** 2021-06-01

**Authors:** Simone Miller, Daniela Diers, Michael Jungheim, Cornelia Schnittger, Hans Jörg Stürenburg, Martin Ptok

**Affiliations:** 1 Department of Phoniatrics and Pediatric Audiology, Hannover Medical School, Hannover, Germany; 2 Klinikum Region Hannover GmbH, KRH Geriatrie Langenhagen, Germany; 3 Department of Neurology, Klinik Niedersachsen, Erwin Röver GmbH und Co. KG, Bad Nenndorf, Germany

**Keywords:** neuromuscular electric stimulation, dysphagia, swallowing, deglutition, deglutition disorders

## Abstract

**Background:** Previous results of clinical studies suggest that neuromuscular electrostimulation (NMES) therapy, especially in combination with traditional dysphagia therapy, may be helpful in patients with neurogenic swallowing disorders. In these studies, repetitive application of a rectangular current impulse was used to increase muscle strength of the anterior neck. However, according to sports physiological findings, an increase of muscle strength can be better achieved by using different NMES stimulation protocols, e.g. KOTS. The aim of the translational investigator-initiated, non-commercial pilot study presented here was to provide data and insights for the planning of subsequent phase II and III studies on the effectiveness of such stimulation protocols in dysphagia therapy.

**Methods:** 30 post-stroke patients with oropharyngeal dysphagia were included in this prospective pilot study and randomly allocated to either neuromuscular electrostimulation (NMES) or sham stimulation in combination with traditional dysphagia therapy (TDT), a pre- and post-therapeutic fiberoptic-endoscopic evaluation of swallowing (FEES) with the Dysphagia Outcome and Severity Scale (DOSS) (primary outcome measure), Secretion Scale by Murray, Penetration and Aspiration Scale (PAS) and throat clearance (TC) abilities. Recruitment rate, interrater comparison and number of relevant adverse events were recorded as metadata.

**Results:** Despite a recruiting time of over 24 months, only twelve patients could be included. Moreover, clinical data indicated a significant variance of clinical pictures. Significant differences in verum versus sham therapy were not observed. DOSS values in both study groups showed general improvements at the end of the trial. Interrater reliability was low. No adverse events were reported.

**Discussion:** When planning further dysphagia therapy studies, it must be taken into account that it can be problematic to recruit sufficiently large study collectives within an appropriate study period. This is especially important since a possible additional benefit of NMES to TDT is probably rather small or may only occur in certain deficit constellations. The low interrater reliability observed here must be improved by appropriate training measures. Fortunately, no relevant undesirable side effects occurred. This could have a positive effect on the acceptance of volunteers to participate in the study.

## Zusammenfassung

**Hintergrund:** Bisherige Ergebnisse klinischer Studien deuten darauf hin, dass eine neuromuskuläre Elektrostimulationstherapie (NMES) insbesondere in Kombination mit einer traditionellen Dysphagietherapie (TDT) bei Patient_innen mit neurogenen Schluckstörungen hilfreich sein könnte. Bei diesen Studien sollten durch repetitive Applikation eines Rechteckstrom-Impulses Muskeln der vorderen Halsseite gestärkt werden. Eine Intensivierung der Muskelkraft ist allerdings nach sportphysiologischen Erkenntnissen durch Anwendung von anderen NMES-Stimulationsprotokollen, z.B. nach KOTS, besser zu erzielen. Ziel der hier vorgestellten translationalen Prüfer-initiierten, nicht kommerziellen Studie war es, Daten und Erkenntnisse für die Planung anschließender Phase-II- und -III-Studien zur Frage der Effektivität alternativer Stimulationsprotokolle in der Dysphagietherapie zu gewinnen.

**Methoden:** Gemäß Studienprotokoll sollten 30 Patient_innen mit einer Dysphagie nach einem Apoplex untersucht werden. Die Zuordnung zu Studienarmen dieser prospektiven Pilotstudie (NMES nach KOTS oder Scheinstimulation (sham), immer zusätzlich zu TDT) erfolgte randomisiert. Bei allen Patient_innen wurde prä- und posttherapeutisch eine fiberoptisch-endoskopische Evaluation des Schluckaktes (FEES) in Kombination mit der Dysphagia Outcome and Severity Scale (DOSS), der Sekretbeurteilungsskala nach Murray, der Penetrations-/Aspirationsskala (PAS) nach Rosenbek und der Bewertung der Reinigungsfunktion durchgeführt. Die TDT + NMES oder + sham erfolgte über einen Zeitraum von 4 Wochen, 5 Tage pro Woche, zweimal täglich für jeweils 20 min. Als Metadaten wurden Rekrutierungsrate, Interraterrealibilität und Anzahl relevanter Vorkommnisse erfasst.

**Ergebnisse:** Nach der festgelegten Laufzeit über 24 Monate konnten nur zwölf Patient_innen rekrutiert werden, die jeweiligen klinischen Bilder waren zudem inhomogen. Signifikante Unterschiede der Verum- versus Scheintherapie zeigten sich nicht. Lediglich die DOSS-Werte zeigten in beiden Studiengruppen generelle Verbesserungen nach der Therapiephase, allerdings war die Interraterreliabilität niedrig. Unerwünschte Nebenwirkungen wurden nicht berichtet.

**Diskussion:** Für die Planung weiterer Dysphagie-Therapiestudien muss berücksichtigt werden, dass es problematisch sein kann, ausreichend große Studienkollektive innerhalb eines angemessenen Studienzeitraums zu rekrutieren, insbesondere, da ein eventueller Zusatznutzen einer NMES zur TDT vermutlich eher klein ist oder eventuell nur bei bestimmten Defizitkonstellationen auftritt. Die hier beobachtete niedrige Interraterreliabilität muss durch geeignete Schulungsmaßnahmen verbessert werden. Erfreulicherweise traten keine relevanten unerwünschten Nebenwirkungen auf. Dies könnte sich positiv auf die Akzeptanz von Probandinnen und Probanden zur Studienteilnahme auswirken.

## Introduction

Swallowing of solid and liquid food is a highly complex and partially automated mechanism which has social and vital impact. Dysphagia, the impairment of swallowing, might cause e.g. malnutrition, dehydration, social isolation, and in case of an insufficient protection of the lower airways also aspiration pneumonia or death [1].

Stroke is one of the major factors that can impair swallowing. The incidence of strokes in Germany is as high as 262,425 per year [2]. The prevalence of dysphagia after stroke is reported at around 8.1–45.3%, depending on the severity and brain region being affected [3], [4]. During the first 3 weeks (acute till post-acute phase) following the event, the rate of spontaneous remission is very high, but a large amount of patients still present with dysphagia after 12 months, during the chronic stage of disease [4], [5]. Thus, there is an increasing number of patients needing treatment.

Traditional dysphagia therapy represents the general choice of therapy for post-stroke dysphagia in order to retrain lost functions or acquire adaptive strategies to ensure safety of swallowing [5], [6]. Safe and sufficient food intake and hydration should therefore always represent the aim of dysphagia treatment. Unfortunately, TDT alone does not always provide sufficient success in the treatment of dysphagia. Meanwhile the use of neuromuscular electrostimulation (NMES) is well established in the rehabilitative context and has recently also become the focus of interest as an alternative or adjunct treatment in dysphagia therapy [7]. Whereas still controversial with regard to dysphagia caused by stroke, results imply that NMES combined with and without TDT as an adjunct therapy might be more effective than traditional dysphagia therapy alone in the treatment of dysphagia [7], [8], [9].

Clinical studies examining the benefits of NMES in stroke patients made use of a device emitting a square symmetrical biphasic waveform with interphase interval pulse. This class II device passed an FDA 510 (k) request (K153224), regulation number 890.5850. The indications for use claim that a muscle re-education by application of external stimulation to the muscles is necessary for pharyngeal contraction [10]. Interestingly, the predicate mentioned is another device by the same company intended for use for the same purpose and the predicate for this grandfather device are devices for general muscle stimulation [11].

Several studies indicate that other forms of electric pulses might be superior to rectangular pulses for increasing muscle strength [12], [13]. Thus, it seemed reasonable to investigate if such stimulation protocols are useful for muscle strengthening therapy in post-stroke dysphagic patients. Here, we examined a protocol using an electric current of medium frequency, called KOTS (named after the Russian inventor) or Russian Technique. This protocol was initially used to improve muscle strength in Olympic athletes [13].

For future design of a phase II clinical study and eventually initiating the development of an approvable new therapy device, a phase I study was started. It was expected to gain insight into potential pitfalls to encounter when initiating a phase II study and to obtain preliminary, but not confirmatory results regarding the efficiency of applying a KOTS protocol.

## Materials and methods

### Design

This research was conducted to unveil potential pitfalls in studying the effects of specific NMES protocols. We designed a translational investigator-initiated phase I prospective randomized multicenter pilot trial. The protocol included stroke patients who were allocated randomly to either an NMES (KOTS) stimulation protocol or sham stimulation. All patients received +/– NMES in addition to traditional dysphagia therapy by trained speech therapists. The time period was 2;4 years from January 2013 until April 2015.

### Patients

Male and female patients with post-stroke mild to severe oropharyngeal dysphagia (DOSS 1–5, cerebrovascular insult) during the post-acute phase (from around 2 weeks till 4 months following the event [14]) were included in this study. They were treated in a rehabilitative clinic (RC) setting at one of two participating clinics. A balanced sex ratio was not considered necessary since no gender- or sex-specific differences were expected. The study was approved by the institution’s ethics committee (#6068/2012). Informed consent has been obtained from all participants before any study-related activity was started. Non-consent, pregnancy, diseases of the upper esophageal sphincter, tumors, pacemakers, cranial stimulators as well as metal implants in the head and neck represented exclusion criteria. The study was performed in accordance with the Declaration of Helsinki, Good Clinical Practice, and applicable regulatory requirements, e.g. regulation (EU) 2016/679.

### Randomization and blinding

During this prospective and randomized pilot study, dysphagic patients received TDT in combination with NMES or sham stimulation over a period of four weeks. Randomization of the protocols was insured by the draw of a concealed envelope. The random allocation sequence was generated by the authors of this article, whereas therapy was implemented by the rehabilitation facilities’ speech and language therapists. Patients were blinded towards the status of therapy. All other investigators were blinded to the condition until study-related activities were finished and baseline and final examinations analyzed. 

### Pre- and post-treatment examination

Fiberoptic endoscopic evaluation of swallowing (FEES) in combination with the following protocols was performed for baseline and final examinations [15], [16], [17]:

- Dysphagia Outcome and Severity Scale (DOSS),
- Secretion Scale by Murray (SS-Murray),
- Penetration and Aspiration Scale (PAS),
- throat clearance (TC).

Each swallowing examination video, recorded by two speech pathologists, was evaluated by two highly qualified and experienced phoniatricians, who were blinded to study activities and patients’ conditions. Pre- and post-therapy (baseline vs. final examination) DOSS values were used for primary analysis. Secondary analysis included descriptive analysis of secretion scale as well as the Penetration and Aspiration Scale by Rosenbek.

### Intervention procedure

Therapies were performed in cooperation with two rehabilitation facilities. Both facilities treat patients of post-acute stroke. Speech and language therapists had been trained to perform the desired NMES protocol previously. NMES sessions were performed 5 days per week, twice per day for 20 min each. Therapy besides NMES was performed by speech pathologists and was based on TDT.

### Device and neuromuscular electrical stimulation protocol KOTS

To apply the preselected NMES (KOTS) protocol, a device (batch) was developed according to the needs of this study (similar to “investigation device exemption” [18], or Art. 82 MDR 2017/245, which will come into force next year). The manufacturer (PHYSIOMED Electromedizin AG, Schnaittach, Germany) of this device is certified according to EN ISO 13485:2016. Where applicable, the device met the requirements of electronic security according to IEC 60601-1 rev. 4 with the exception of IEC 60601-2-10, 201.7.9.2.101h.

During NMES therapy, one pair of electrodes is placed submentally over the digastricus, geniohyoid and mylohyoid muscle, which together elevate the os hyoideum in an anterior-superior direction. A paralaryngeal placement of a second pair of electrodes aims to stimulate the inner larynx muscles (cricoarytaenoid lateral muscle, arytaenoid transverse muscle, thyroarytaenoid muscle and vocal muscle). Furthermore, an electric current of medium frequency (basic frequency=2.5 kHz, modulation frequency=50 Hz, pulse duration=2 ms, pulse shape=rectangle) is used in order to stimulate muscle activation accordingly and to penetrate deeper into the tissue. Intensity was set individually to above motor threshold for stimulation and below motor threshold (at 1 mA) for sham stimulation.

### Traditional dysphagia treatment

Therapeutic contents of TDT were based on FEES findings and clinical swallowing examination by the swallowing therapist. Functional dysphagia therapy [19] is based on three components: restitution, compensation and adaptation. All components were used in the context of the study. The most frequently applied methods were thermal stimulation, posture adaptation, lingual/larynx-motional exercises and mobilization in the field of restitution. The most commonly used compensatory strategies were chin tuck and saliva swallows. Consistency modification of foods and liquids (adaptation) were implemented for all patients.

### Protocol identifying procedural difficulties and deficiencies

This protocol aimed to identify difficulties and deficiencies. Moreover, this protocol addressed safety issues (e.g. adverse effects progressing dysphagia, skin burn, irritating electric leak current, induction of muscle cramps). Secondly, it addressed procedural issues, i.e. all formal aspects of conducting the study (e.g. patient recruiting, obtaining informed consent, difficulties and deficiencies analyzing results).

### Statistical analysis

A statistical analysis was performed using IBM SPSS Statistics version 23. Differences between final examination and baseline examination were tested using the Wilcoxon signed-rank tests. The test was used to test differences between two consecutive measurements. Differences were considered significant when the p-value was <0.05.

## Results

### Conceptual results

The term *conceptual results* will refer to findings relating to the compliance of results according to the study plan and planned analysis scheme.

#### Patient recruitment

Recruiting patients turned out to be a major problem. Although two stroke rehabilitation centers were involved and the study time frame was initially considered sufficient, not 30 patients as planned but only 12 patients could be included (see patient characteristics, Table 1 (Tab. 1)). Individual patient records proofed all patients to be suffering from stroke; however, causes of stroke and stroke health-related consequences differed significantly. One major reason for this low recruitment number were difficulties in obtaining the informed consent from patients. One patient who had started study activities had to be excluded due to early discharge and missing final examination.

#### Safety issues

Throughout the study, no serious adverse effects were observed. Moreover, no skin irritation by heat or electric current was reported. Targeted muscles did not respond to NMES with muscle cramps or spasms.

#### Deficiencies in the analysis of results

In order to assess the performance of the KOTS protocol preliminarily, the swallowing ability by means of measuring 5 semi-objective parameters previously described as useful in the evaluation of dysphagia (DOSS, SS-Murray, GC-Murray, PAS, TC [17]) were investigated (Table 2 (Tab. 2), Table 3 (Tab. 3)). One major obstacle for subsequent data analysis was an undue high number of missing data. Secondary analysis revealed that missing data was due to susceptibility to errors of certain parameters, as the quality of the video and/or anatomic structures. Besides missing data, an inappropriate interrater reliability (DOSS) was recorded.

### Procedural results

The term *procedural results* will refer to empirical data obtained with respect to clinical results employing KOTS.

11 patients (3 women, 8 men, mean age: 76 years) with dysphagia resulting from cerebrovascular insults were included in the study. One patient (female) had to be excluded due to early discharge and missing final examination. 7 patients underwent stimulation, while 4 patients received sham treatment.

#### Primary parameter

Differences between both groups were tested for significance using the Mann Whitney U test. For both investigators (I1 and I2), the stimulation and the sham group showed no significant differences (both p=0.527) between the scores of baseline DOSS. Both groups showed improvements from baseline to final examination (Table 2 (Tab. 2)). In the stimulation group (1), 4 patients showed better results in the DOSS values for the final than baseline examination, and 3 or 4 patients (depending on the investigator) for the sham group (0). The shared increase in the sham group results in 3 or 5 points (depending on the investigator) and 6 or 9 points (depending on the investigator) for the stimulation group.

In the stimulation group, 1 patient’s swallowing abilities deteriorated during treatment and 2 patients showed no change at all. In the sham group, 0 or 1 (depending on the investigator) patient’s swallowing abilities worsened and 1 or 0 (depending on the investigator) patients showed no change after treatment. No other harms or unintended effects were found.

The Wilcoxon signed-rank test shows no significant effects for DOSS for both groups (Table 4 (Tab. 4)). However, the slope of the mean values expressed at a percentage shows that the stimulation group had a little more improvement in the DOSS values for both investigators (I1: 19%; I2: 33%) than the sham group (I1: 15%; I2: 31%).

#### Secondary parameters

Regarding PAS values, all patients showed a substantial increase after treatment in the overall values. According to investigator 1, patients in the stimulation group had an overall increase of 4 points, whereas sham patients showed an increase of 6 points. Investigator 2 stated a larger increase in the stimulation group by 10 points, compared to 7 points in the sham group. Hence, the stimulation group showed no improvements for investigator 1 (p=0.357) or for investigator 2 (p=0.109). The sham group shows no improvements for investigator 1 (p=0.357) or for investigator 2 (p=0.066) either.

Regarding the secretion status, both investigators fully agreed on the trend each patient took. The cumulative score of the stim group adds up to an improvement of 5 or 6 points (depending on the investigator). In the sham group 1 (I1) or 2 (I2), patients showed improvements, 2 patients showed no changes, and 0 (I1) or 1 (I2) patient deteriorated regarding the secretion status between investigations. The overall score of the sham group adds up to an improvement of 3 (I1) or 0 (I2) points. Neither group presents with an effect of statistical significance (stimulation group: I1 p=0.129, I2 p=0.098; sham group: I1 p=0.180, I2 p=1.000).

Clearance results showed no changes between baseline and final investigations in 5 patients (investigator 1) and 1 patient (investigator 2) of the stimulation group. 2 or 5 patients (depending on the investigator) showed an improvement of 1 to 2 points. One patient was stated to show a deterioration of 1 point for investigator 2. Differences between baseline and final examinations in the stimulation group measured p=0.564 for investigator 1 and p=0.096 for investigator 2. In the sham group, 3 or 1 patient(s) showed improvements and 1 or 3 patient(s) showed no changes after treatment. Mean values in the sham group measured p=0.102 for investigator 1 and p=0.317 for investigator 2.

## Discussion

Persisting dysphagia following stroke still presents a therapeutic challenge. TDT based on training of weak muscles, changing swallowing behavior, positioning or diet modification has proven to be of some use; however, the quest for innovative and effective therapeutic measures has to go on. NMES has the potential to be a useful adjunct to TDT, and may be applied in order to e.g. prevent muscle atrophy after denervation or synkinesis after nerve injury, or to foster regeneration of muscle-nerve units after nerve trauma and to increase muscle strength [7]. The latter is addressed with current (and FDA-cleared) medical devices. Assuming that strengthening muscle force is helpful for some dysphagic patients, it was reasoned that an NMES protocol not based on rectangular pulses might be even more effective.

In order to answer such a research question, i.e. whether NMES is useful in treating dysphagia, several steps are necessary, such as designing a prototype device, preclinical studies, and phase I through to phase III studies. A certified medical device manufacturer was able to supply a device batch that had been produced according to those specific specifications, namely integrating a KOTS protocol. A preclinical study was not deemed to be necessary. At this point, a phase I study had to be carried out.

### Conceptual results and pitfalls

The results from this study provided insights with regard to necessary improvements, especially for designing a subsequent phase II study (Table 5 (Tab. 5)):

- Despite the seemingly long time period and despite the fact that two rehabilitation centers were involved, not enough patients could be included in the study. This calls for either an even longer time frame, or the involvement of more RCs. The latter might be more appropriate. A rough estimate shows that based on the figures presented here, approximately 5 RCs must participate in order to recruit 30 patients, or 16 in case 100 patients are needed over the same period of time.
- Problems were experienced with obtaining informed consent, due to the mental state of the participants. Certainly, there is no simple solution to overcome this problem.
- Missing data may be an obstacle not only encountered here. It is possible that the instruction given to the speech and language therapists was not sufficient. Furthermore, it might be more helpful if the phoniatrician analyzes the swallowing capability live and directly, and not as recorded video material.
- Somewhat surprisingly, an unsatisfactorily low interrater reliability was found, despite the fact that both raters at that stage had already evaluated more than 2000 FEES. As a consequence, it might be helpful to train even highly experienced raters in a sense that more congruent results are achieved, even in questionable findings.

### Procedural results

In general, one has to keep in mind that this study was not intended to produce confirmatory results. The results of this pilot study suggest no statistically significant differences between NMES and sham stimulation regarding the DOSS outcomes after the intervention period. Very interestingly, both groups (sham and stimulation) showed overall improvements in swallowing abilities as measured by DOSS after treatment. This implies that both treatment forms are generally effective. No untreated control group was investigated, since this would have been unethical. Though spontaneous remission was not controlled for, it is not as relevant during this phase as it would occur earlier after the stroke, during the acute phase. With an overall difference, however, of 3 or 4 points (depending on the investigator) and visible in the percentages (slope of the mean values), the improvements in relation with NMES seem substantially higher than improvements caused by TDT alone. Previous research also has shown that NMES, combined with TDT, offers better and faster therapy effects [7], [20], [21].

The results reflected by PAS differ from the results seen for DOSS in that the difference between the two study groups is much more blurred. Quite a large number of patients showed no change in penetration or aspiration categories as measured by PAS. Rather than reflecting no improvement at all, this could be due to the fact that PAS is not a “steady scale” as such, where a higher number (e.g. 5) would necessarily always reflect worse abilities than the lower number (e.g. 4).

Nevertheless, the overall increase in both groups is found to be rather large.

DOSS evaluates and therewith reflects the overall swallowing competence, whereas most of the scales investigated secondarily focus on individual performances during swallowing, and do not necessarily have to correlate strongly with each other or with DOSS.

The parameter “glottic closure” was initially evaluated during this study, but results were not carried through to the results section. With regard to glottic closure, one has to keep in mind that this is quite a complex task for stroke patients, as it requires task understanding and execution of a voluntary task. Both components are often impaired after stroke. The results that were achieved might therefore not truly reflect the glottic closure capability. Furthermore there are a lot of missing values which make comparison rather difficult or impossible. This parameter therefore did not prove to be the most reliable parameter and does not lend itself to be included in future studies investigating swallowing abilities in stroke patients.

For the secretion status and the efficiency of clearance there is no significant improvement before and after the treatment of both groups. However, the values of I2 for the stimulation group compared to the values of the sham group tend to be closer to the level of significance. These scales might correlate more strongly with DOSS. 

Furthermore, subjective dimensions like quality of life were not considered in this study. They would certainly represent an important factor in dysphagia therapy and should be considered for future investigations.

The two study groups compared in this study were statistically similar to baseline. However, a limitation of this study is that the groups differ in numbers because there are more patients in the stimulation group and the overall number of patients was small.

Fortunately, no adverse events were reported. Although these are not valid results due to the low number of patients included, the absence of adverse events is encouraging and may be helpful in obtaining informed consent.

## Conclusion

The data presented here point to several pitfalls that must be overcome in future studies: it may be difficult to include a sufficient number of patients, parameters must be carefully chosen, small effect sizes need to be considered, and it is recommendable that more than two raters participate. Furthermore, action should be taken to avoid missing data.

The exploratory data show no differences in therapy effects between stimulation and sham group for two investigators. Results also suggest, however, that the selected stimulation protocol combined with TDT as well as TDT alone is effective in patients presenting with post-stroke (severe to mild) dysphagia. The technique of NMES is well tolerated.

Since several unforeseen problems were encountered, new study protocols must be designed in future phase II studies.

## Notes

### Ethical approval

The study was approved by the institution’s ethics committee (#6068/2012) and was performed in accordance with the Declaration of Helsinki, Good Clinical Practice, and applicable regulatory requirements, e.g. regulation (EU) 2016/679.

### Informed consent

Informed consent has been obtained from all patients before any study-related activity was started.

### Authors’ contributions

Simone Miller and Daniela Diers have shared first authorship.

### Funding

As part of a third party funded project, this work was funded by the AiF Project GmbH of the BMWi (Germany’s Federal Ministry for Economic Affairs and Energy), KF 2448401FR9.

### Competing interests

The authors declare that they have no competing interests.

## Figures and Tables

**Table 1:**
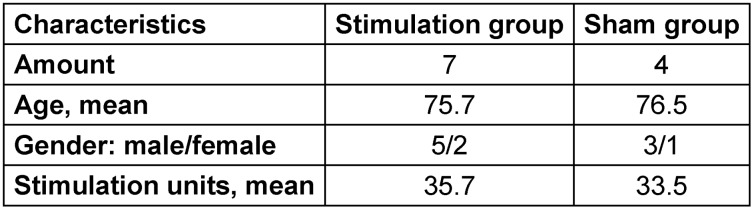
Patient characteristics

**Table 2:**
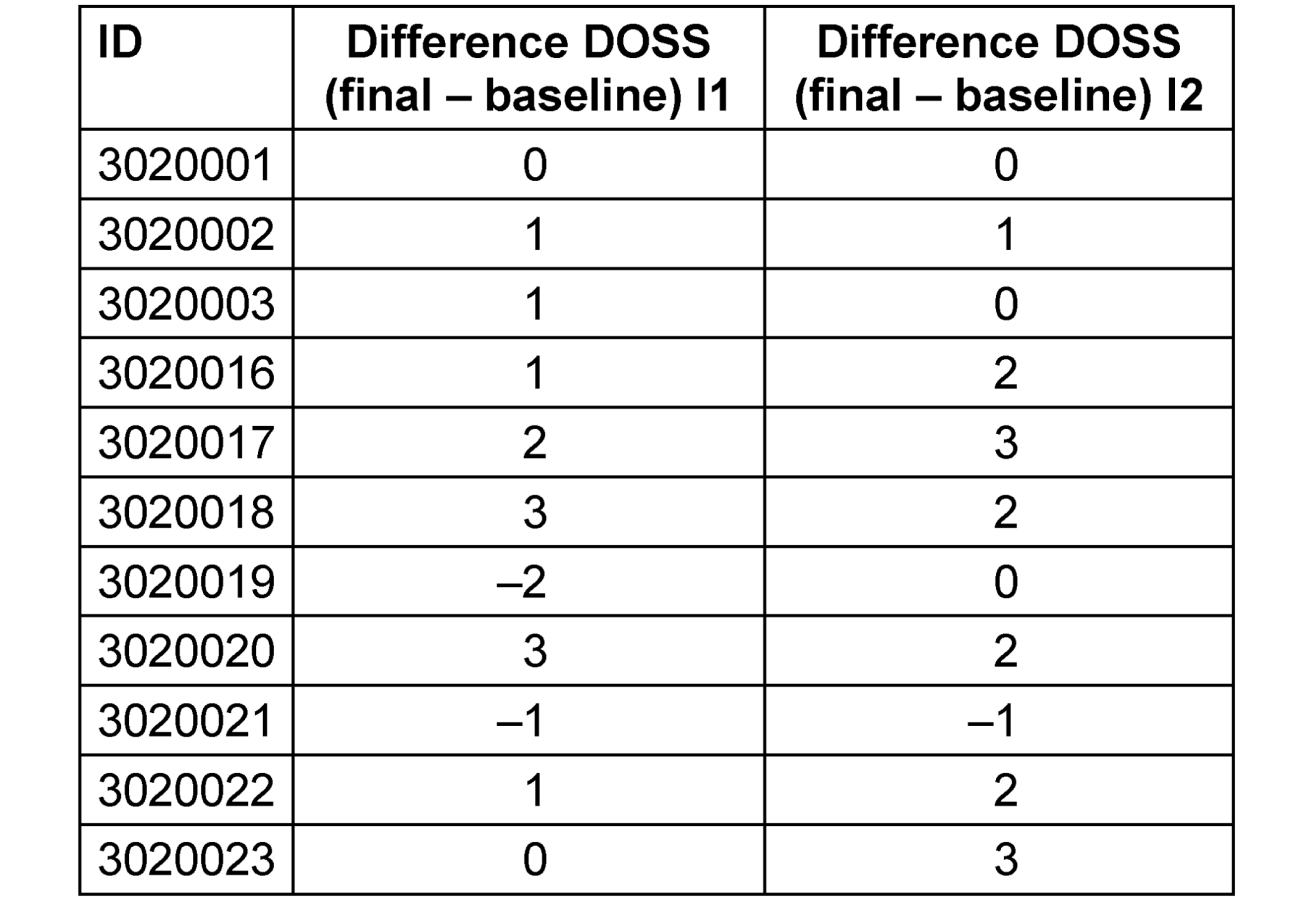
Overview of baseline versus final examination Dysphagia Outcome and Severity Scale (DOSS) results of all patients by two investigators (I1 and I2)

**Table 3:**
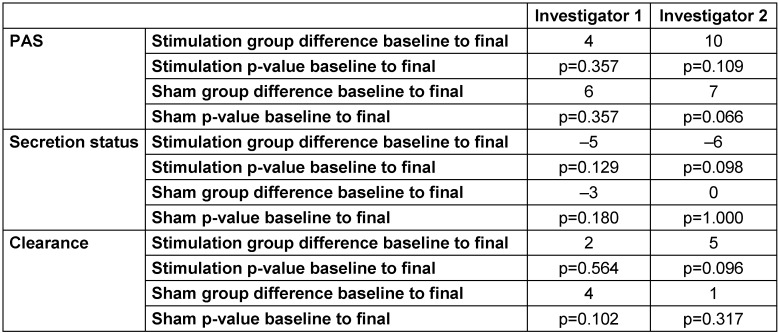
Overview results secondary parameters

**Table 4:**
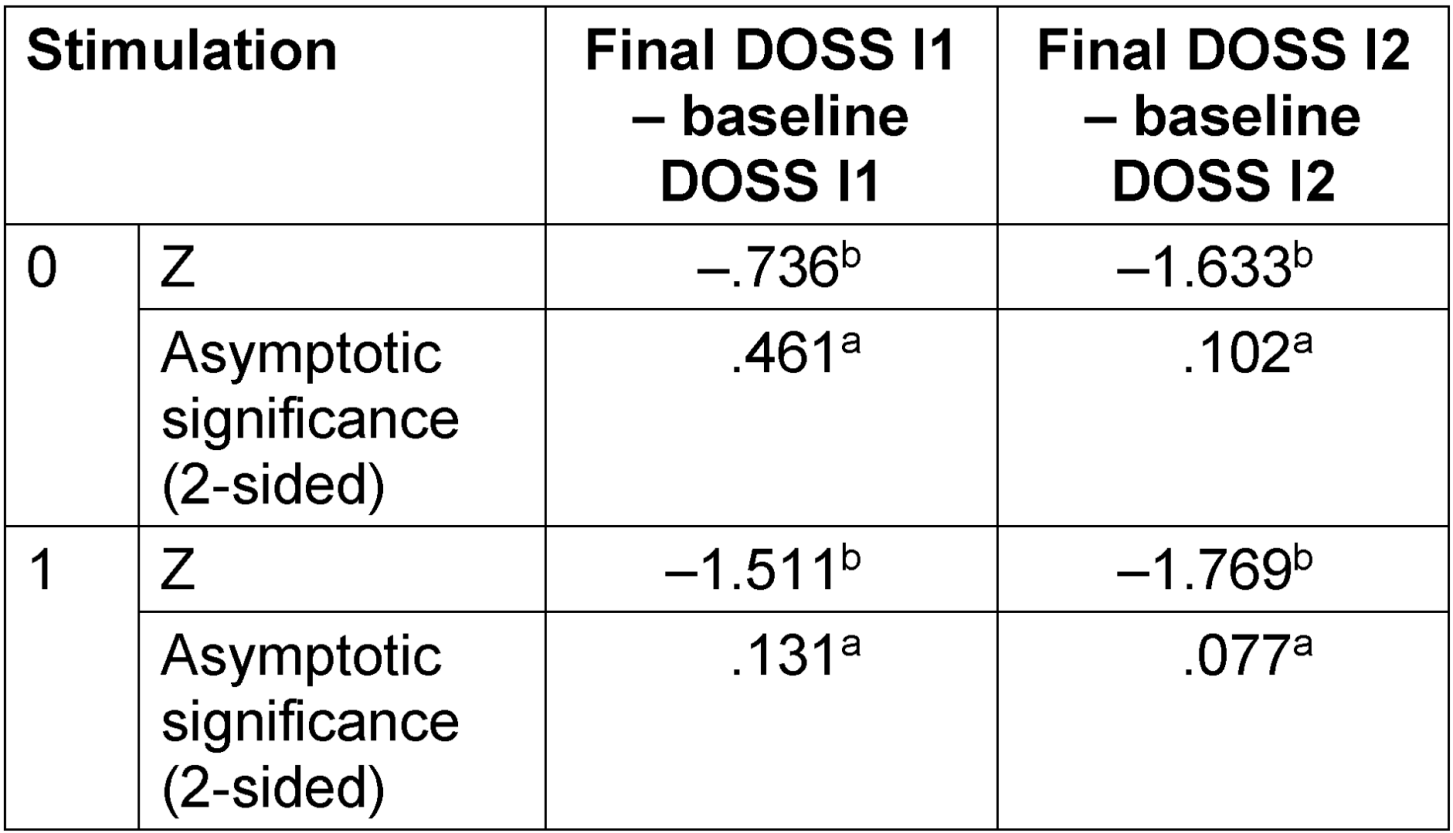
Wilcoxon signed-rank tests; a Wilcoxon test, based on negative ranks of baseline versus final examination Dysphagia Outcome and Severity Scale (DOSS) results of all patients by two investigators (I1 and I2)

**Table 5:**
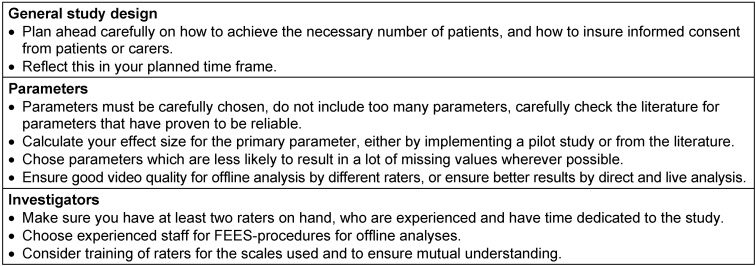
Overview of occurring pitfalls and how to avoid them
